# Endangered Atlantic Sturgeon in the New York Wind Energy Area: implications of future development in an offshore wind energy site

**DOI:** 10.1038/s41598-019-48818-6

**Published:** 2019-08-27

**Authors:** Evan Corey Ingram, Robert M. Cerrato, Keith J. Dunton, Michael G. Frisk

**Affiliations:** 1 School of Marine and Atmospheric Sciences, Stony Brook University, Stony Brook, New York, 11794 USA; 2 Department of Biology, Monmouth University, West Long Branch, New Jersey, 07764 USA

**Keywords:** Animal migration, Behavioural ecology, Animal behaviour, Animal migration, Marine biology

## Abstract

Imminent development of offshore wind farms on the outer continental shelf of the United States has led to significant concerns for marine wildlife. The scarcity of empirical data regarding fish species that may utilize development sites, further compounded by the novelty of the technology and inherent difficulty of conducting offshore research, make identification and assessment of potential stressors to species of concern problematic. However, there is broad potential to mitigate putatively negative impacts to seasonal migrants during the exploration and construction phases. The goal of this study was to establish baseline information on endangered Atlantic Sturgeon in the New York Wind Energy Area (NY WEA), a future offshore development site. Passive acoustic transceivers equipped with acoustic release mechanisms were used to monitor the movements of tagged fish in the NY WEA from November 2016 through February 2018 and resulted in detections of 181 unique individuals throughout the site. Detections were highly seasonal and peaked from November through January. Conversely, fish were relatively uncommon or entirely absent during the summer months (July–September). Generalized additive models indicated that predictable transitions between coastal and offshore habitat were associated with long-term environmental cues and localized estuarine conditions, specifically the interaction between photoperiod and river temperature. These insights into the ecology of marine-resident Atlantic Sturgeon are crucial for both defining monitoring parameters and guiding threat assessments in offshore waters and represent an important initial step towards quantitatively evaluating Atlantic Sturgeon at a scale relevant to future development.

## Introduction

Offshore wind endeavors are increasingly being regarded as readily-available sources of renewable energy^1–5^. Favorable domestic policy, coupled with the rapid development and maturation of overseas wind energy markets during the last two decades, has created impetus for the exploitation of offshore wind energy resources in the US^6–9^. The majority of near-term activities are concentrated in the Northeast and Mid-Atlantic regions of the US where a number of projects in federal waters of the Outer Continental Shelf (OCS) have been proposed or are in the planning stages, dependent on clean energy goals and initiatives of individual states.

Imminent development of the OCS has led to concerns about the potential for offshore wind farms to negatively impact marine ecosystems and fauna^10–13^. The novelty of the technology and the inherent difficulty of conducting offshore research make identification and assessment of potential stressors to marine wildlife problematic^12,14^. Impacts of operational offshore wind farms—both positive and negative—are often locally influenced and contingent on specific site, species’ spatial and temporal distribution, and management objectives; however, there is broad potential to mitigate negative impacts during the exploration and construction phases through spatial and temporal considerations, particularly for migratory species that may only utilize a development site on a seasonal basis^15–17^.

Studies regarding the impacts of offshore wind development have largely focused on marine mammals and seabirds^18–21^ and there is a relative scarcity of information regarding marine fish species^22–25^. The lack of empirical data, particularly for commercially important and federally protected marine fish species, underscores the need for targeted research to better quantify the likely effects of offshore wind energy development^14,16,26^. Baseline data collection and modeling are critical for future decision making and regulation in wind energy areas and are especially important when designing impact assessments for species of concern with limited or no existing information on offshore distribution or abundance^16^.

The federally protected Atlantic Sturgeon (*Acipenser oxyrinchus oxyrinchus*) is a species of concern that may occupy marine habitat allocated for future offshore wind development. Atlantic Sturgeon are an anadromous, long-lived species with a broad distribution along the Atlantic Coast of the US. Atlantic Sturgeon are highly migratory and exhibit a complex life-history that is dependent on access to both freshwater and marine environments, contingent on life-stage. Although adults undertake obligatory migrations into natal river systems for spawning purposes, the majority of the late-juvenile and adult life-stages are spent in coastal marine waters^27^. Delayed sexual maturity and the subsequent increase in reproductive output that occurs at later ages indicate that a focus on the reduction of mortality in the marine resident life-stages of Atlantic Sturgeon is necessary to restore depleted populations^28–30^. Despite this, basic knowledge of Atlantic Sturgeon in marine waters is limited and, consequently, the identity and magnitude of current and emerging threats are often difficult to assess.

In the New York Bight (NYB), limited scientific and commercial fisheries data suggest that Atlantic Sturgeon spend significant time in near-coastal marine waters^31–36^. Documented movements and aggregation areas of Atlantic Sturgeon in the NYB are concentrated along the coasts of New York and New Jersey and have been observed within 13-km of the shoreline^33^. Commercial fisheries bycatch of Atlantic Sturgeon has been observed in offshore areas that are beyond recognized aggregation sites^34,37^. The Hudson River stock makes up a large part of the coastal bycatch of Atlantic Sturgeon in the NYB (42.2–46.3%^38^); however, the mixing of coast-wide genetic stocks that occurs in marine waters emphasizes the potential for emerging, localized threats (i.e., offshore wind farm development) to affect multiple stocks^39,40^.

The New York Wind Energy Area (NY WEA; Equinor, Lease OCS-A 0512), located between Long Island and the coast of New Jersey, is an offshore wind-lease area in the exploration and site assessment phase of development that will ostensibly host active construction in the near-future; as such, the area provides a unique opportunity to study the spatial and temporal trends of Atlantic Sturgeon in a future offshore wind energy site. Because effective management of Atlantic Sturgeon requires an understanding of marine movements and habitat use, the identification of the temporal and spatial trends of Atlantic Sturgeon in the NY WEA will provide important baseline information regarding habitat use and environmental preferences in offshore marine waters. These data are critical to informing management decisions in the NY WEA and are necessary to inform required statutory consultations and impact assessments under the Endangered Species Act and the Outer Continental Shelf Lands Act^41,42^. Consequently, the goal of this study was to establish threshold information on endangered Atlantic Sturgeon in a future offshore wind energy site, with the specific objectives of identifying: (1) spatiotemporal trends in offshore occurrence; (2) residency in the NY WEA; and (3) environmental predictors of offshore movement.

## Methods

### Study site

**Figure 1:**
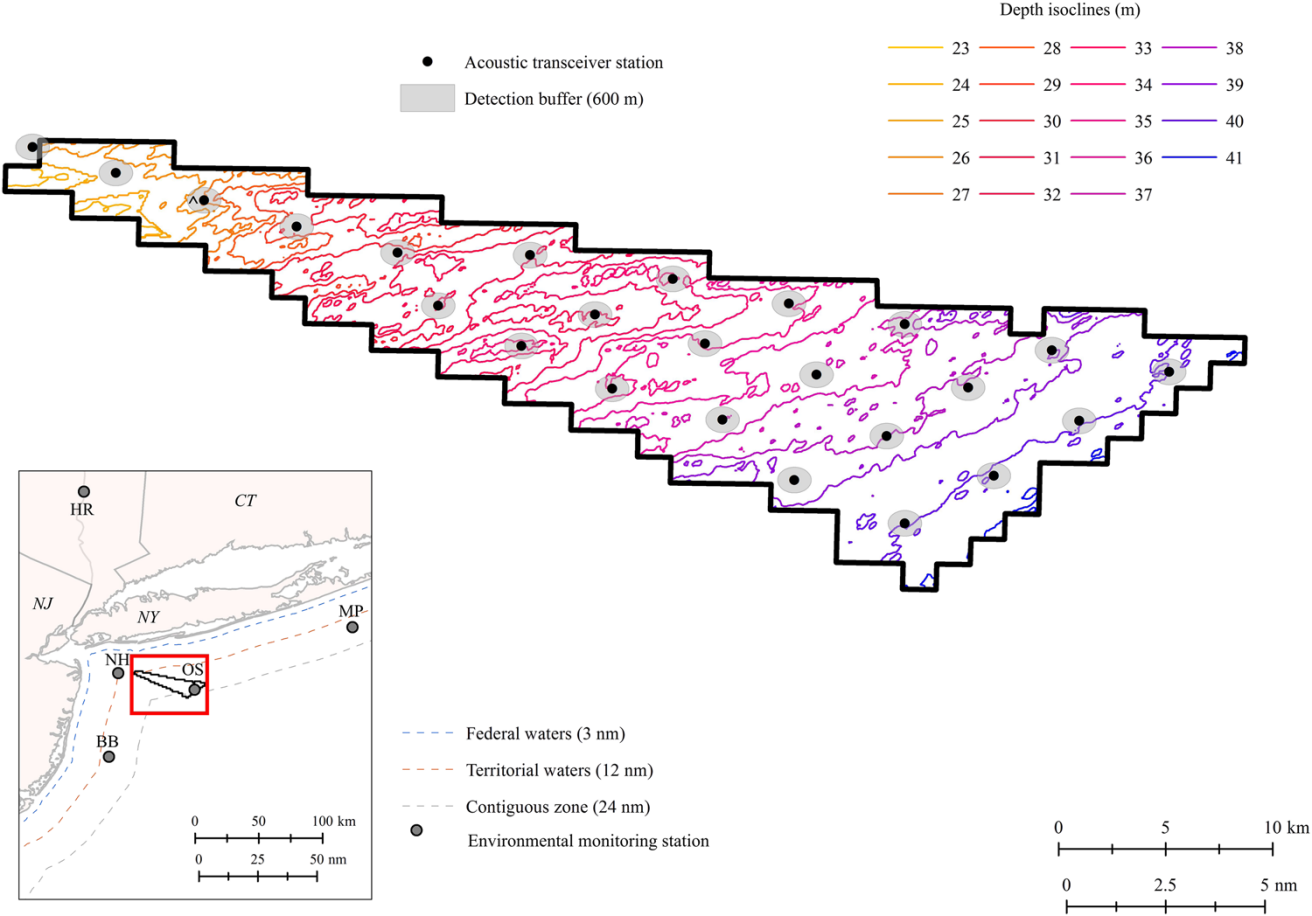
Map of the New York Wind Energy Area study site (NY WEA; Equinor, Lease OCS-A 0512) and inset showing the relative location in federal waters of the Atlantic Ocean off the coast of New York and New Jersey. Transceiver station locations, assumed 600-m detection radii, and 1-m depth isoclines within the NY WEA are indicated. The transceiver array operated throughout the entire course of the study (November 10, 2016–February 5, 2018) with the exception of a single station indicated by (^) which was not recovered during the final download cruise; data for this station were unavailable for the period of August 4, 2017–February 5, 2018. Locations of relevant environmental monitoring stations are shown (NH = Entrance to New York Harbor, NOAA NDBC Station 44065; BB = Coastal Barnegat Bay, NOAA NDBC Station 44091; MP = Coastal Montauk Point, NOAA National NDBC Station 44017; OS = Offshore New York, NOAA NDBC Station 44025; HR = Hudson River below Poughkeepsie, New York, USGS Gauging Station 01372058).

The NY WEA encompasses approximately 79,350 acres of offshore euhaline habitat in the NYB region of the western Atlantic Ocean, east of the Hudson Shelf Valley (Fig. 1). Located in federal waters off the coast of New York and New Jersey, the site extends 22–48 km (11.5–24.0 nm) southeast of Long Island, New York, and forms an expanding wedge positioned between the Ambrose to Nantucket (eastbound) and Hudson Canyon to Ambrose (northwest-bound) traffic lanes. The Cholera Bank feature, located adjacent to the westernmost end of the NY WEA, was removed from the original lease area^43^. Water depths in the NY WEA range from 23 to 41 m and generally increase away from shore in a southeasterly direction^44^. The bottom habitat is characterized as relatively flat and primarily composed of sandy sediments, although isolated patches of gravelly, muddy sand exist^44,45^. Seasonal fluctuations are strong in the study area and bottom temperatures range from 2 to 22 °C. Thermal stratification between bottom and surface waters generally occurs from April to August, with turnover during the fall months^44^.

### Fish sampling

All methods for the capture and handling of Atlantic Sturgeon in this study were performed in accordance with relevant guidelines and regulations and were authorized by the National Marine Fisheries Service (NMFS; Endangered Species Permits 16422 and 20351), New York State Department of Environmental Conservation (Endangered/Threatened Species Scientific License 336), and Stony Brook University’s (SBU) Institutional Animal Care and Use Committee (IRB-1022451–4).

Marine resident Atlantic Sturgeon [i.e., juvenile (500–1,000 mm fork length [FL]), sub-adult (1,000–1,300 mm FL), and adult (>1,300 mm FL) life-stages, based on NMFS permitting definitions] were opportunistically sampled in May 2016–2018 and October 2017 during targeted research tows aboard the RV Seawolf. Tows occurred peripheral to the study site and targeted known marine aggregations located off the Rockaway Peninsula, New York; sampling relied on previously described trawl gear^33,34^. Tows occurred in relatively shallow waters (8–20 m) at speeds of 3.0–3.5 knots for short durations (5–15 min) in order to maximize capture efficiency while minimizing the stress placed on captured fish.

Atlantic Sturgeon were immediately sorted from the catch and transferred to an onboard live-well where they were allowed to recover. All fish were then examined for internal and external tags. If none was found, a passive integrated transponder tag was inserted into the body musculature beneath the fourth dorsal scute and an external dart tag was inserted near the dorsal fin base. Measurements of total length, FL, and weight were recorded. Age-at-capture was estimated using the von Bertalanffy growth function^46^ and parameter estimates for Atlantic Sturgeon from the NYB distinct population segment (*L*_∞_ = 278.87, *K* = 0.057, *t*_0_ = −1.27^47^). A uniquely-coded VEMCO (Halifax, Nova Scotia) acoustic transmitter (V-16 6 H; 69 kHz; tag delay 70–150 s; estimated battery life = 3,650 d) was then surgically implanted into each individual fish^48,49^. Following surgeries, fish were returned to the live-well and monitored for 5–10 min until they had fully recovered before being released near their original capture site.

### Passive acoustic telemetry

In November 2016, a stationary array consisting of 24 acoustic-release transceivers (VEMCO VR2AR) with omnidirectional hydrophones was deployed throughout the NY WEA to monitor movements of acoustically tagged fish (Fig. 1). Submerged transceivers were attached to anchored buoys and deployed so that they were suspended approximately 2 m from the seabed. Transceivers were placed in a grid pattern with nearest-adjacent transceiver stations located less than 4 km apart [mean (range) = 3.43 (2.87–3.94) km]. Preliminary range testing at transceivers was accomplished using a transponding hydrophone (VEMCO VR100) and revealed an average maximum detection radius of approximately 600 m (range = 200–1,000 m), depending on sea state. Previous studies utilizing acoustic receiver arrays in coastal waters have assumed a 600 m detection radius based on high detection rates of tags at similar ranges (e.g., ~65% at 600 m and a maximum range of 1,400 m^50^). Although the western-most transceiver was not directly positioned in the NY WEA, the assumed detection radius of 600 m overlapped with the study site and was therefore included in analyses.

An onboard tracking receiver and transponding hydrophone permitted surface communication with individual transceivers; acoustic-release and recovery of transceivers was facilitated through remote detachment of the transceiver from a sacrificial anchor during download and maintenance. Transceivers with acoustic release mechanisms allowed for deeper and longer deployments in offshore marine waters without the need for diver retrieval. Transceiver array download and maintenance cruises occurred in August 2017 and February 2018. The transceiver array operated throughout the entire course of the study (November 10, 2016–February 5, 2018) with the exception of a single station which was not recovered during the final download cruise; data for this station were unavailable for the period of August 4, 2017–February 5, 2018 (Fig. 1).

Telemetry data were carefully reviewed to identify and remove any spurious detections that were obvious based on the spatial and temporal chronology of individual fish^51^. Data management and analysis were primarily performed in R^52^. Additional detections of Atlantic Sturgeon that were tagged by SBU researchers during previous sampling efforts were included in analyses to increase the robustness of the study (~380 previously tagged Atlantic Sturgeon assumed at-large in fall 2016^53^).

### Environmental data

Potential environmental predictors were compiled from a variety of sources and matched to daily unique counts of Atlantic Sturgeon detections in the NY WEA. Environmental variables were selected based on putative biological significance to Atlantic Sturgeon as reported in previous studies as well as availability and completeness of datasets during the time-period of interest. Daily photoperiod and moon-fraction illumination were calculated using the R package “suncalc”^52^. Sea surface temperatures were compiled from environmental monitoring stations maintained by the National Data Buoy Center (NDBC) in nearshore-coastal and offshore waters, including: the entrance to New York Harbor (NOAA NDBC Station 44065), coastal Barnegat Bay, New Jersey (NOAA NDBC Station 44091), coastal Montauk Point, New York (NOAA National NDBC Station 44017), and offshore New York (NOAA NDBC Station 44025; Fig. 1). Hudson River environmental data, including temperature and discharge, were obtained from the United States Geological Survey (USGS) gauging station located at river kilometer (rkm) 115 in the lower Hudson River below Poughkeepsie, New York (USGS 01372058; Fig. 1). Average hourly bottom temperatures in the NY WEA were compiled from transceiver records; from these, monthly point values at transceiver locations were interpolated onto a raster surface using an inverse distance weighted technique in ArcGIS to simplify visual comparison of monthly temperatures in the NY WEA. Because anadromous Atlantic Sturgeon migrate between heterogeneous environments, pair-wise differences between water temperatures were also examined to explore potential triggers of movement between habitats.

### Residency and movement

Periods of residency and movement were calculated using the behavioral event qualifier in the R package “V-Track”^53–55^. A residence event was defined as a minimum of two successive detections of an individual at a single transceiver station over a minimum period of two hrs. Residence events were terminated by either a detection of the individual on another transceiver station or a period of 12 hrs without detection (i.e., time-out period). Movement events were defined as non-residence events (i.e., movements of an individual between two transceivers) and were limited to non-residence events of less than five days. Rate of movement (ROM) was calculated using a transceiver-distance matrix that assumed direct distance movements and a 600 m detection radius for each transceiver. When available, calculations were aided by detections of Atlantic Sturgeon from cooperative arrays located outside of the study area.

### Generalized additive models

Unique daily counts of Atlantic Sturgeon in the NY WEA were modeled using generalized additive models (GAMs)^56^. All GAMs were built in the R package “mgcv”^57^ using thin plate splines^58,59^. A log-link function with a quasi-Poisson error distribution was used to account for overdispersion in the count data. Independence of daily counts was assumed to be valid based on previously reported movement rates of Atlantic Sturgeon that suggest that daily mixing can occur. The initial model included photoperiod, moon-fraction illuminated, water temperatures, and discharge, and considered complexity up to first-order interactions. Stepwise backwards elimination of explanatory variables was performed to determine a minimally adequate model using minimization of the generalized cross validation (GCV) criterion to guide the process. AIC is not available for quasi-Poisson models, and GCV is an acceptable alternative in subset regression^60,61^. During model selection, GCV scores were compared with and without an explanatory variable to determine which terms to remove. Smoothing functions were replaced by linear terms if the estimated degrees of freedom approached a value of one, suggesting that a smooth was essentially a straight line^62^. Because the water temperatures for the New York Harbor and Hudson River estuary were highly correlated predictors (*r*^2^* = *0.91), only the latter was used to capture potential cues in river temperature associated with Atlantic Sturgeon movement. As a test of the quality of the selection process, the final and full models were compared using an F-ratio test to determine if the final (i.e., selected) model explained significantly less of the residual error compared to the full model^62^. A significant result would indicate that the final model explained less of the residual error than the full model and that the final model was under-parameterized.

## Results

### Fish sampling and acoustic telemetry

Atlantic Sturgeon (n = 133) were captured via targeted bottom trawling and tagged with acoustic transmitters during sampling cruises in May 2016 (n = 40), May 2017 (n = 81), and October 2017 (n = 12). The size range of the tagged fish was 619 to 2,050 mm FL, with a mean FL of 855 mm. Tagged fish were representative of juvenile (n = 114), sub-adult (n = 17), and adult (n = 2) life-stages, with age-at-capture estimates ranging from 4 to 28 years. Detections of Atlantic Sturgeon tagged during this study as well as at-large fish tagged by SBU researchers during previous sampling efforts were included in analyses to increase the robustness and scope of the study^53^.

**Figure 2:**
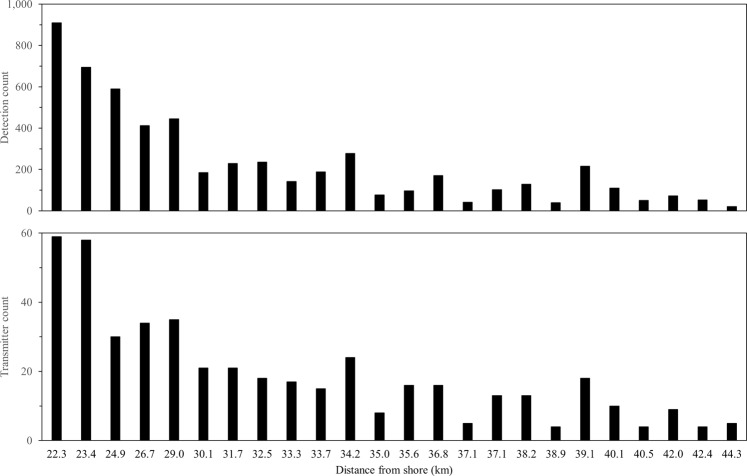
Detection count (top panel) and unique transmitter count (bottom panel) of Atlantic Sturgeon detected on acoustic transceivers in the New York Wind Energy Area study site (Equinor, Lease OCS-A 0512). Transceivers are represented by increasing distance from shore; note that intervals are not equal.

Telemetry data indicated that Atlantic Sturgeon were present in the NY WEA during array operation. Total confirmed detections for Atlantic Sturgeon ranged from 1 to 310 detections per individual, with a total of 5,490 valid detections of 181 unique individuals. Detections in the NY WEA were representative of Atlantic Sturgeon tagged during the study (n = 39; 1,028 detections; Table 1) as well as at-large Atlantic Sturgeon tagged by SBU researchers during previous sampling efforts (n = 142; 4,462 detections; Supplementary Table S1). Atlantic Sturgeon occurred throughout the study site and were detected on all transceivers in the array (Fig. 2); importantly, Atlantic Sturgeon were observed on the most distal transceiver station, located 44.3 km offshore (21 total detections of 5 unique fish). Total counts and detections of unique fish were highest nearer to shore and appeared to decrease with distance from shore. Counts at each station ranged between 21–909 total detections and 4–59 unique detections of Atlantic Sturgeon.

**Table 1:** Biological data associated with Atlantic Sturgeon tagged with acoustic transmitters May 4, 2016–October 11, 2017 that were subsequently detected in the New York Wind Energy Area study site (Equinor, Lease OCS-A 0512). Age-at-capture for individual fish was estimated using the von Bertalanffy growth function^46^ and parameter estimates for Atlantic Sturgeon from the New York Bight distinct population segment (*L*_∞_ = 278.87, *K* = 0.057, *t*_0_ = −1.27)^47^.

Identifier	Release date	Fork length (mm)	Total length (mm)	Weight (kg)	Age	Life-stage	Detections
ATS-01	May 4, 2016	920	1,060	8.26	7	J	53
ATS-02	May 4, 2016	780	910	4.30	5	J	14
ATS-03	May 4, 2016	770	860	4.42	5	J	9
ATS-04	May 4, 2016	940	1,060	7.41	7	J	15
ATS-05	May 4, 2016	1,030	1,130	10.64	8	SA	13
ATS-06	May 4, 2016	907	1,033	5.91	6	J	4
ATS-07	May 4, 2016	790	920	4.34	5	J	26
ATS-08	May 4, 2016	920	1,040	6.60	7	J	24
ATS-09	May 4, 2016	720	820	3.18	5	J	79
ATS-10	May 4, 2016	790	890	4.39	5	J	70
ATS-11	May 4, 2016	720	844	3.37	5	J	64
ATS-12	May 4, 2016	1,153	1,311	13.37	9	SA	12
ATS-13	May 4, 2016	944	1,070	7.01	7	J	5
ATS-14	May 4, 2016	926	1,041	7.28	7	J	7
ATS-15	May 4, 2016	859	977	6.65	6	J	24
ATS-16	May 4, 2016	911	985	6.11	7	J	11
ATS-17	May 4, 2016	944	1,091	7.98	7	J	41
ATS-18	May 4, 2016	1,009	1,126	5.50	8	SA	32
ATS-19	May 4, 2016	755	878	3.29	5	J	9
ATS-20	May 4, 2016	1,082	1,219	11.16	9	SA	36
ATS-21	May 9, 2017	723	826	3.55	5	J	2
ATS-22	May 9, 2017	955	1,100	9.19	7	J	13
ATS-23	May 9, 2017	890	1,014	6.40	6	J	33
ATS-24	May 9, 2017	704	757	2.91	4	J	2
ATS-25	May 9, 2017	974	1,108	8.84	7	J	30
ATS-26	May 9, 2017	1,620	1,832	38.12	17	A	11
ATS-27	May 9, 2017	708	792	3.40	4	J	7
ATS-28	May 9, 2017	1,017	1,160	10.88	8	SA	40
ATS-29	May 10, 2017	1,059	1,234	11.04	8	SA	64
ATS-30	May 10, 2017	872	934	6.52	6	J	35
ATS-31	May 10, 2017	1,124	1,279	13.92	9	SA	9
ATS-32	May 10, 2017	765	904	3.84	5	J	80
ATS-33	May 11, 2017	843	952	4.98	6	J	37
ATS-34	May 11, 2017	963	1,201	8.50	7	J	4
ATS-35	May 11, 2017	789	921	4.28	5	J	7
ATS-36	May 11, 2017	880	1,017	6.24	6	J	18
ATS-37	October 11, 2017	917	1,055	5.11	7	J	15
ATS-38	October 11, 2017	896	1,031	5.47	6	J	32
ATS-39	October 11, 2017	1,071	1,245	10.37	8	SA	41

Note: J = juvenile (500–1,000 mm FL); SA = sub-adult (1,000–1,300 mm FL); A = adult (>1,300 mm FL); based on National Marine Fisheries Service permitting definitions.

**Figure 3:**
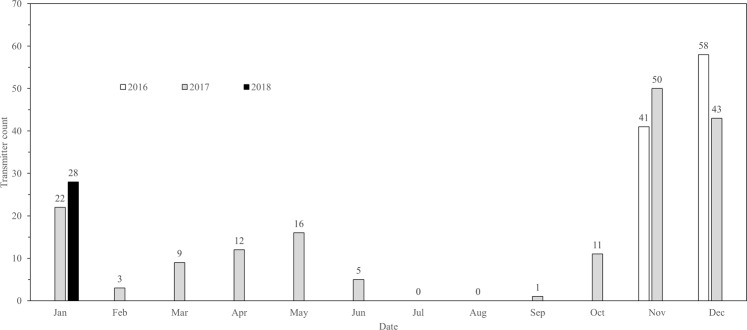
Monthly counts of unique Atlantic Sturgeon detected on acoustic transceivers in the New York Wind Energy Area study site (Equinor, Lease OCS-A 0512) from November 2016 through January 2018.

**Figure 4:**
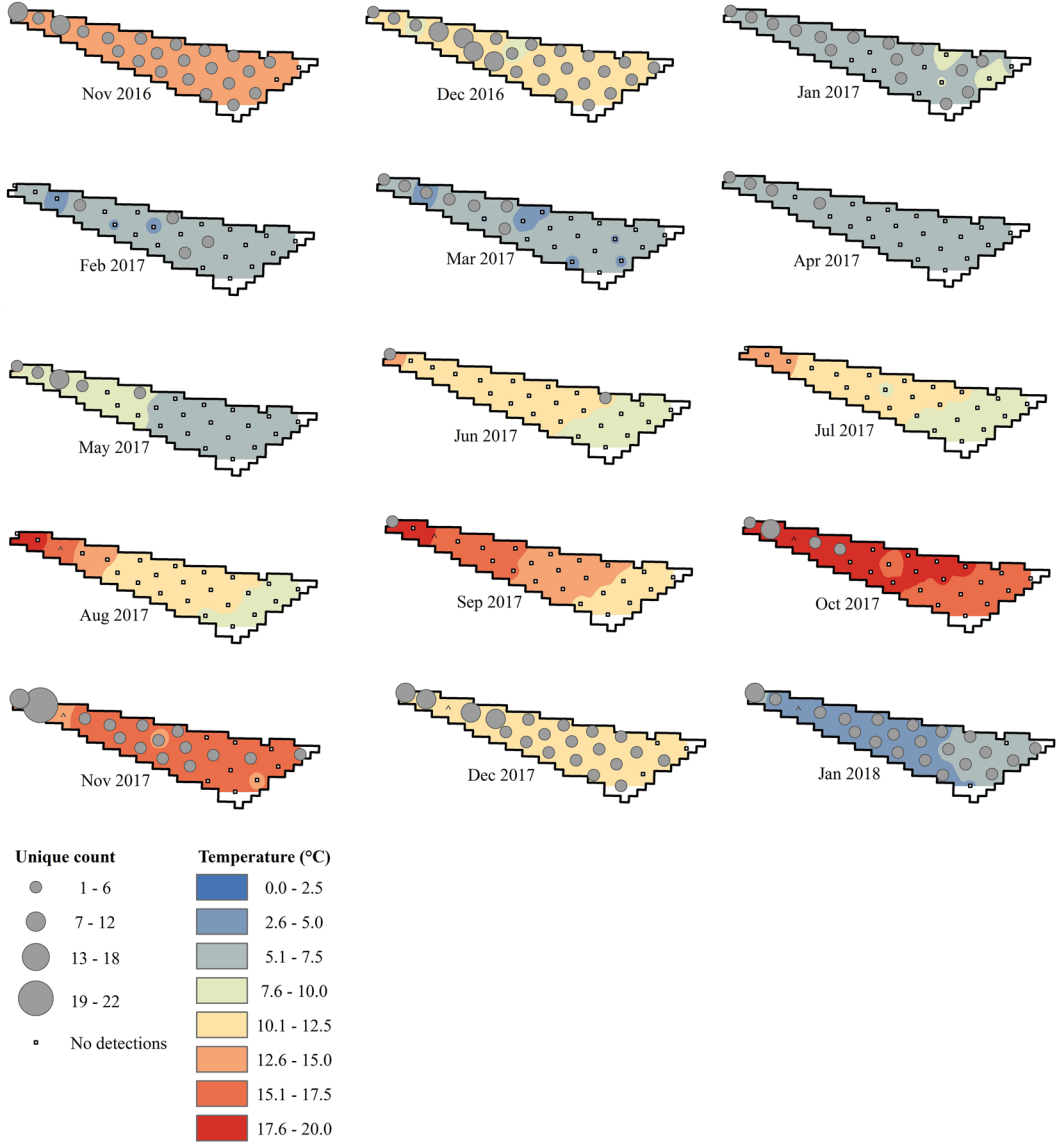
Monthly counts of unique Atlantic Sturgeon (represented by graduated symbols) detected at unique acoustic transceiver stations in the New York Wind Energy Area study site (Equinor, Lease OCS-A 0512) from November 2016 through January 2018. Monthly point values of average bottom temperature were compiled from transceiver metadata. The transceiver array operated throughout the entire course of the study with the exception of a single station indicated by (^) which was not recovered during the final download cruise; data for this station were unavailable for the months of August 2017–February 2018.

Atlantic Sturgeon were regularly detected in the NY WEA throughout the study period and 55 individuals were observed in the site during multiple years. During 2016 and 2018, transceivers were only operational in the study site for a short period (November 10–December 31, 2016 and January 1–February 5, 2018) but 87 unique fish (2,098 detections) and 30 unique fish (761 detections) were detected, respectively. In 2017, the array was operational for the entire year and 126 Atlantic Sturgeon (2,631 detections) were observed in the study site. Monthly counts of individuals in the NY WEA were highest during the months of November, December, and January, and peaked in December 2016 (n = 58) (Figs 3 and 4). Importantly, two years of data are available for these months and similar abundances were observed for both datasets (e.g., relatively high abundances of Atlantic Sturgeon occurred during November and December 2016–2017 and January 2017–2018). Atlantic Sturgeon were relatively uncommon (i.e., <2 individuals detected) or entirely absent from the NY WEA during July, August, and September (Figs 3 and 4).

Within the NY WEA, both temporal and spatial variation in unique counts of Atlantic Sturgeon were observed (Fig. 4). The majority of individual fish were detected on transceivers located nearer to shore except during months of relatively high abundance when fish were more widely distributed throughout the array. The highest observed abundance of Atlantic Sturgeon at a single station (n = 22; 23.4 km from shore) occurred in November 2017; however, during this month individuals were observed in the study site >40 km from shore, demonstrating the wide distribution of fish in the NY WEA. During the months of December and January, fish were present and evenly distributed across the majority of transceivers in the NY WEA and in December 2016 Atlantic Sturgeon were detected on all transceivers in the array. Average monthly bottom temperatures observed in the study site ranged from 2.1 to 19.0 °C, with local minimum temperatures in February–March and local maximum temperatures in August–October (Fig 4). Evident temperature stratification between bottom and surface waters in the study site was observed April–November (Supplementary Fig. S2).

**Figure 5:**
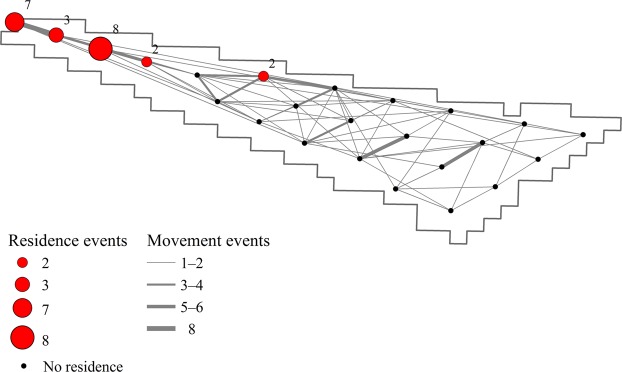
Atlantic Sturgeon residence and non-residence events at transceiver stations in the New York Wind Energy Area study site (Equinor, Lease OCS-A 0512) from November 10, 2016–February 5, 2018. Residence events are defined as a minimum of two successive detections of an individual at a single transceiver station over a minimum period of two hrs. Residence events are completed by either a detection of the individual on another transceiver station or a period of 12 hrs without detection. Non-residence events (i.e., movements of an individual between two transceivers) were limited to periods of less than five days.

### Residency and movement

Residence events at individual transceiver stations in the NY WEA were uncommon (n = 22) and were only observed on five transceivers during the study period, with the majority of residence behaviors associated with near-shore stations in the NY WEA (Fig. 5). The station with the highest number of observed residence events (n = 8) was located 24.9 km from shore, and no residence events were observed beyond 30.1 km from shore. Residence events were of short duration [mean (SD) = 10.1 (14.0) hrs; range = 2.1–70.1 hrs] and the maximum ROM observed between stations was 0.86 m/s, although slower rates were common [mean (SD) = 0.31 (0.20) m/s]. By assuming the maximum observed ROM of 0.86 m/s and maximum straight-line distance of 40.6 km between stations from the transceiver-distance matrix, the minimum transit time for an Atlantic Sturgeon through the NY WEA at its longest point was estimated to be 13.1 hrs. This suggests that daily mixing could occur and, consequently, that the assumption of independence for daily counts used to model the data was valid.

**Figure 6:**
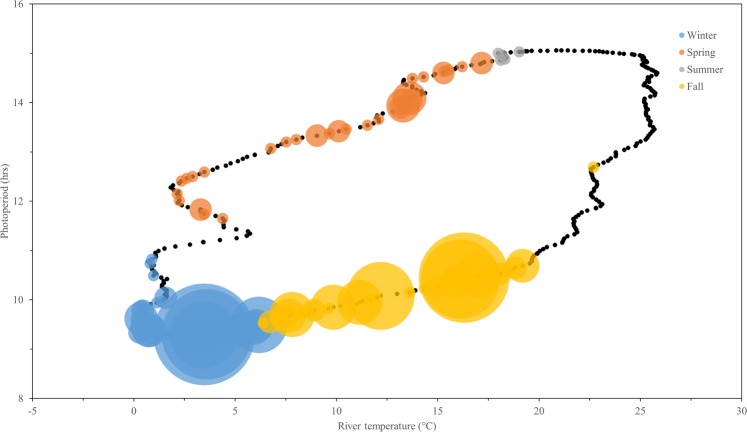
Interaction between photoperiod and water temperature in the Hudson River estuary, New York, during 2017. Circle size represents the daily unique count of Atlantic Sturgeon in the New York Wind Energy Area study site (Equinor, Lease OCS-A 0512). Water temperature is from the USGS Gauging Station 01372058 at Hudson River below Poughkeepsie, New York. Winter = December 1–February 28; Spring = March 1–May 31; Summer = June 1–August 31; Fall = September 1–November 28.

### Generalized additive model

The final, simplified GAM model contained a smooth term for the interaction between Hudson River estuary water temperature and photoperiod and a linear term for river discharge as predictors of unique daily counts of Atlantic Sturgeon in the NY WEA:

$${\rm{U}}{\rm{D}}{\rm{C}}\sim s({{\rm{H}}{\rm{R}}}_{{\rm{t}}{\rm{e}}{\rm{m}}{\rm{p}}},\,{\rm{P}})+{{\rm{H}}{\rm{R}}}_{{\rm{d}}{\rm{i}}{\rm{s}}{\rm{c}}{\rm{h}}{\rm{a}}{\rm{r}}{\rm{g}}{\rm{e}}}$$

where UDC is unique daily count of Atlantic Sturgeon in the NY WEA, HR_temp_ is daily mean water temperature (°C) in the lower Hudson River, HR_discharge_ is daily mean discharge (ft^3^/s) in the lower Hudson River, P is daily photoperiod (hrs), and *s*() indicates a smoother was used. The final model explained 61.0% of the deviance and had a GCV score of 0.9407. No significant difference was found between the final and full model (*F*_7.04,409.23_ = 1.58, *p* = 0.1389), providing evidence that the selection process identified an appropriately complex model.

Exploration of the relationship between daily abundance of Atlantic Sturgeon in the NY WEA and the two-way interaction term above allows for clarification of the model structure (Fig. 6). Both temperature and photoperiod terms are cyclical (i.e., the same value can occur during a positive or negative trend) and out of phase. As water temperatures in the Hudson River decreased below 20 °C during the fall months along with decreasing daily photoperiod, daily counts of Atlantic Sturgeon in the NY WEA were observed to increase and reached a maximum as water temperature in the Hudson River approached its annual minimum (~0.0 °C). Conversely, as water temperatures in the Hudson River increased during the spring and into the summer months along with increasing daily photoperiod, detections of Atlantic Sturgeon in the NY WEA decreased and fish were entirely absent when temperature was at its summer maximum. While water temperature in the Hudson River was strongly correlated to photoperiod (*r*^2^ = 0.49), there was a notable temporal lag of ~ 35 days, presumably because of the high heat capacity of water. During this lag in the late summer, Atlantic Sturgeon were not detected in the NY WEA when temperatures in the river were near their maximum, despite the decreasing trend in photoperiod. The response curve for the linear discharge term had a clear, decreasing trend, and suggests that high daily abundance of Atlantic Sturgeon in the NY WEA was more likely in the fall and winter during periods of low river discharge (less than 20,000 ft^3^/s; Supplementary Fig. S3).

## Discussion

This study provides benchmark information regarding the incidence and seasonality of marine-resident Atlantic Sturgeon use of offshore waters in the NY WEA and, importantly, identifies potential estuarine drivers of offshore occurrence. With the development of offshore wind energy in the United States comes the recognized need to prioritize offshore research and monitoring in the context of ecological risk assessment and mitigation; here, we establish effective baseline criteria regarding spatial and temporal trends of Atlantic Sturgeon within the putative area of effect—an obligatory prerequisite for evaluating the impact of activities during all stages of offshore wind energy development. Furthermore, this study demonstrates the effectiveness of acoustic-release transceivers for discerning cryptic behaviors of species of concern in an offshore wind energy site through the targeted monitoring of acoustically tagged individuals.

Overall, the results of this study suggest that offshore distribution of Atlantic Sturgeon in the NY WEA is highly seasonal. Observations of fish on the acoustic array broadly corroborate and expand the current knowledge of marine movements in the mid-Atlantic Bight^31–34,37,38^. However, the fine-scale documentation of Atlantic Sturgeon trends in the NY WEA and the identification of putative migratory pathways beyond the extent of traditional survey data may have important implications on species conservation. Consistent spatial and temporal trends of Atlantic Sturgeon occurrence were evident from telemetry data and indicated expansion into deeper, offshore waters of the NY WEA during the fall and winter months. High incidences of Atlantic Sturgeon during the winter were observed in both years, with a marked increase in unique counts and total detections in November and December. The absence of Atlantic Sturgeon in the NY WEA during the summer months, particularly from June through September, suggests a putative shift to nearshore habitat and corresponds with periods of known-residence in shallow, coastal waters that are associated with juvenile and sub-adult aggregations as well as adult spawning migrations^29,55,63–65^.

Despite the recent focus on coastal research, as well as the consequent designation of in-river critical habitat (n = 31 Critical Habitat Units)^66^, the offshore trends and distribution of Atlantic Sturgeon remain relatively unknown and the biological and physical features essential for their conservation in marine habitats have not been identified. The results of this study should help define monitoring parameters in offshore waters and guide future assessments in marine wind energy areas. An important finding from the telemetry data was that Atlantic Sturgeon used the majority of habitat available to them within the NY WEA. Individual counts and total detections of Atlantic Sturgeon were highest on transceivers located in shallow waters and exhibited a generally decreasing trend with increased depth and distance from shore. Although limited observations of adult Atlantic Sturgeon have been described on shelf waters at depths of up to 40 m^32^, the ubiquity of Atlantic Sturgeon documented throughout the study site was unexpected and suggests that the distribution and habitation of these fish in marine waters is greater than previously assumed. Throughout the range, research is needed to further characterize the extent of the offshore distribution of Atlantic Sturgeon; however, these results demonstrate the importance of targeted studies in marine waters where more traditional survey- or fisheries-dependent methodologies may underestimate habitat use, particularly at intermediate scales or at the boundaries of the known distribution of highly migratory fish.

The influence of environmental factors on Atlantic Sturgeon counts in the NY WEA suggests that transitions between coastal and offshore habitat are predictable and associated with long-term cues and localized estuarine conditions. Anadromous fish populations are known to undertake extensive annual migrations to optimize temporally predictable foraging and spawning conditions^67,68^; these migrations are assumed to be governed, at least in part, by seasonal and ontogenetic responses to complex abiotic factors^67^. In sturgeon species, photoperiod and water temperature (and, to a lesser extent, discharge) are recognized as factors that act to modulate migratory strategies^69,70^. Although the specific mechanisms driving movements and ontogenetic migrations of Atlantic Sturgeon are not well known, clinal variations in abiotic conditions have been broadly associated with temporal distribution and habitat selection^35,51,71^. The final GAM model indicates that a small subset of abiotic factors provide context-dependent cues regarding the timing of offshore migration. Day-length (i.e., photoperiod) is a reliable long-term trigger of migration that is largely autonomous of annual variation in environmental conditions, while both river temperature and discharge provide short-term signals that result from dynamic, localized conditions. Further evaluation of these cues as predictors of offshore migration is necessary on both a regional and coast wide scale; regardless, the identification of specific abiotic factors that trigger migration and, consequently, offshore distribution has important management implications for monitoring and conservation efforts in future wind energy sites.

Movements and distributions of Atlantic Sturgeon in marine habitats are widely acknowledged to be indicative of preferential selection for covarying environmental properties that are encountered *in-situ*^35,72^. Interestingly, although we considered various marine predictors from both within and outside of the study area, terms used as proxies of marine environmental conditions were not represented in the final model. The elimination of these terms during model selection was informative and suggests that in-river conditions have a significantly greater influence on annual offshore transitions than those encountered while at sea, at least on the scale considered in this study. Likewise, coastal or latitudinal movements, which might otherwise have been inferred from the influence of pair-wise differences in nearby marine conditions (e.g., Barnegat Bay, New Jersey, or Montauk Point, New York), were not indicated by the final model. Although telemetry detections from this study were limited and do not provide evidence of relocations outside of the NY WEA, the seasonal occurrence of marine-migrant Atlantic Sturgeon in near-shore and coastal waters is well documented and corresponds to periods when few or no fish were detected in the study site. In the Hudson River estuary, specifically, marine-migrant life-stages are present from April until the end of November^63–65^ and aggregate in adjacent coastal waters during May, June, September, and October before dispersing^34,35^, which is suggestive of temporal and spatial resource partitioning.

Preferential distribution of Atlantic Sturgeon on bottom types associated with high prey density, most notably sand and gravelly-sand substrates, has been inferred based on commercial bycatch and stomach content analysis^31,73^ but a clear linkage between foraging behavior and resource utilization in marine waters has yet to be determined. The presence of Atlantic Sturgeon over sand-dominated substrates throughout the NY WEA is informative and adds to the literature regarding marine habitat use; however, it does not provide direct evidence of foraging activities in the area. Additional information regarding vertical distributions of Atlantic Sturgeon and, particularly, the relationship between swimming depth and bottom depth could provide further indication as to habitat use^74^; however, the current criteria for defining foraging habitat in marine environments remain largely circumstantial or unknown, and further characterization of ecological correlates of foraging area use with environmental parameters is necessary.

The delineation of behavior modes from telemetry data allows for more informative conclusions regarding perceived habitat selection within the NY WEA as well as explicit guidance for identifying areas of concern at a scale relevant to future development. Over the course of this study, residence events were uncommon and of short duration, despite the use of a relatively non-restrictive time constraint (i.e., minimum residency period of two hrs) to discern any apparent spatial trends in behavior. Importantly, residence behaviors were generally limited to shallow water sites (i.e., depth < 30 m), which suggests the increased potential for negative interactions to occur in these areas during development activities. Site-fidelity of Atlantic Sturgeon in offshore waters is likely highly-variable based on resource availability and, moreover, may occur at a broad spatial scale beyond the scope of this study, as suggested by the migratory life-history of Atlantic Sturgeon and limited observations of mesoscale distribution^32,35^. Individual direct-distance movement rates observed in the NY WEA are comparable to estimates associated with adult foraging behavior in estuarine habitats^75,76^; however, because of the necessary assumption of direct movement, further classification of these behaviors as directional (i.e., transitory) or non-directional (i.e., foraging) is problematic. Regardless, the delineation of sub-seasonal behavior modes is an important step in linking habitat and resource utilization in marine waters.

While biotelemetry itself is not a new technique^77^, the use of acoustic release mechanisms to complement telemetry data collection is a recent development that allows research in deeper offshore areas where conventional array maintenance and data retrieval are problematic^78^. To our knowledge, the current study is the first to use transceivers equipped with acoustic release mechanisms to monitor fish behavior at an offshore wind energy site and may represent a new paradigm for future assessments. Passive acoustic monitoring techniques, coupled with acoustic release technology, provide long-term functionality and situational flexibility as necessary to inform baseline ecological characterization and subsequent ecological impact assessments in offshore wind energy leases. The recent proliferation of long-term acoustic tagging programs along the Atlantic coast of the US not only allows for the possibility of supplemental detections and increased sample size (e.g., the incorporation of previously tagged individuals into the current study) but also illustrates the need for an integrated approach to allow for among-site or comparative analyses. Although the primary focus of our study was to provide baseline pre-construction data, the methodology used could be modified or extended to provide data throughout all phases of development, including informing behavioral changes or fine-scale spatial shifts in distribution of Atlantic Sturgeon over time.

The findings of this study indicate that mitigation of potentially negative impacts of wind energy development, such as multiple-pulse sound sources, is possible through spatial and temporal avoidance during periods of increased Atlantic Sturgeon incidence. Detections of individuals on all transceivers in the study array provide strong evidence that Atlantic Sturgeon use a large amount of the habitat available to them in NY WEA, albeit on a highly seasonal basis. While previous studies have primarily associated the extreme noise from pile-driving during the construction phase with negative impacts to fish assemblages^79,80^, there is still large uncertainty in the literature regarding the physiological effects or behavioral responses of fish to these disturbances. Likewise, data are limited in regards to response distances and areas of potential effect^16^. The low relative abundance of Atlantic Sturgeon in offshore waters of the NY WEA that was observed during the summer months, when sub-adult and adult life-stages were putatively aggregated in riverine or more near-coastal habitats, suggests that the negative impacts of pile-driving activities on Atlantic Sturgeon could be reduced or largely avoided by incorporating data-directed management measures during the planning stages. Conversely, an increased emphasis on impact monitoring is suggested during periods of increased Atlantic Sturgeon abundance during the winter months—particularly November, December, and January—if construction activities cannot be avoided.

From a more general management standpoint, the observed occurrence of Atlantic Sturgeon in offshore waters of the NY WEA underscores the importance of long-term monitoring to recovery efforts, particularly with regard to life-stages and habitats that may be underrepresented in the literature. While considerable research and management attention has recently focused on riverine and coastal waters^81^, empirical evaluations of Atlantic Sturgeon populations in marine waters are inadequate and limited by a lack of basic knowledge. Recent annual survival rate estimates for Atlantic Sturgeon are already below the suggested threshold for recovery^35,82–84^ and, despite a lack of information regarding the magnitude of emerging threats such as offshore wind energy development, it is apparent that even a moderate increase in mortality resulting from anthropogenic sources could negatively impact Atlantic Sturgeon stocks. Further characterization of the broad spatiotemporal trends of Atlantic Sturgeon in offshore waters are necessary to better define monitoring parameters and guide threat assessments in marine wind energy areas; regardless, this study represents an important initial step towards quantitatively evaluating Atlantic Sturgeon in marine waters at a scale relevant to future development.

## Supplementary information

Supplementary Material

## Data Availability

Atlantic Sturgeon telemetry detections used in the current study are archived and publicly viewable on the Harvard Dataverse website: https://dataverse.harvard.edu/. The complete environmental datasets analyzed during the current study are available from the corresponding author on reasonable request.
