# Supplementary material for: Endangered Atlantic Sturgeon in the New York Wind Energy Area: implications of future development in an offshore wind energy site
Source: Sci Rep. 2019 Aug 27;9:12432. doi: 10.1038/s41598-019-48818-6 (PMC6711969; doi:10.1038/s41598-019-48818-6)
Supplement: Supplementary file 1 — Supplementary Material [file 41598_2019_48818_MOESM1_ESM.pdf]

Endangered Atlantic Sturgeon in the New York Wind Energy Area: implications of future development in an offshore wind energy site

Supplementary Information

Evan Corey Ingram[1], Robert M. Cerrato[1], Keith J. Dunton[2], and Michael G. Frisk[1]

[1]School of Marine and Atmospheric Sciences, Stony Brook University, Stony Brook, New York 11794 USA

[2]Department of Biology, Monmouth University, West Long Branch, New Jersey 07764 USA

Supplementary Table S1.—Biological data associated with Atlantic Sturgeon tagged in previous studies with acoustic transmitters by Stony Brook University researchers (n = 142). Shown are fish that were subsequently detected in the New York Wind Energy Area study site (Equinor, Lease OCS-A 0512). Age-at-capture for individual fish was estimated using the von Bertalanffy growth function[1] and parameter estimates for Atlantic Sturgeon from the New York Bight distinct population segment (L_∞ = 278.87, K = 0.057, t_0 = -1.27) [2].

Identifier	Release date	Fork length (mm)	Total length (mm)	Weight (kg)	Age	Life-stage	Detections
SBU-001	May 24, 2011	985	1,129	9.0	7	J	1
SBU-002	May 24, 2011	1,232	1,397	17.6	11	SA	2
SBU-003	Oct 31, 2011	737	872	3.5	5	J	53
SBU-004	Oct 31, 2011	805	947	3.9	5	J	19
SBU-005	Oct 31, 2011	811	969	5.1	5	J	27
SBU-006	Oct 31, 2011	915	1,078	6.4	7	J	53
SBU-007	Oct 31, 2011	940	1,072	6.7	7	J	109
SBU-008	Oct 31, 2011	965	1,130	6.9	7	J	1
SBU-009	Oct 31, 2011	1,033	1,187	9.6	8	SA	15
SBU-010	Oct 31, 2011	1,060	1,247	10.3	8	SA	12
SBU-011	Oct 31, 2011	1,060	1,225	10.5	8	SA	5
SBU-012	Oct 31, 2011	1,070	1,265	10.5	8	SA	11
SBU-013	Oct 31, 2011	1,090	1,295	12.5	9	SA	13
SBU-014	Oct 31, 2011	1,097	1,290	13.6	9	SA	20
SBU-015	Oct 31, 2011	1,125	1,315	12.0	9	SA	18
SBU-016	Oct 31, 2011	1,130	1,285	12.2	9	SA	10
SBU-017	Oct 31, 2011	1,140	1,340	12.2	9	SA	91
SBU-018	Oct 31, 2011	1,160	1,385	13.7	10	SA	16
SBU-019	Oct 31, 2011	1,210	1,409	15.4	10	SA	2
SBU-020	Oct 31, 2011	1,225	1,424	17.3	10	SA	116
SBU-021	Oct 31, 2011	1,810	2,070	58.0	21	A	23
SBU-022	Nov 08, 2011	756	892	3.2	5	J	9
SBU-023	Nov 08, 2011	950	1,110	7.0	7	J	22

SBU-024	Nov 08, 2011	966	1,120	7.3	7	J	14
SBU-025	Nov 08, 2011	1,020	1,150	9.6	8	SA	53
SBU-026	Nov 08, 2011	1,120	1,325	11.6	9	SA	38
SBU-027	Nov 08, 2011	1,155	1,335	13.4	9	SA	5
SBU-028	Nov 08, 2011	1,185	1,380	9.7	10	SA	131
SBU-029	Nov 08, 2011	1,215	1,440	15.6	10	SA	8
SBU-030	Nov 08, 2011	1,340	1,560	20.8	12	A	1
SBU-031	Nov 09, 2011	746	854	3.2	5	J	13
SBU-032	Nov 09, 2011	786	917	3.6	5	J	9
SBU-033	Nov 09, 2011	849	980	5.1	6	J	8
SBU-034	Nov 09, 2011	849	985	5.2	6	J	4
SBU-035	Nov 09, 2011	926	1,065	6.4	7	J	16
SBU-036	Nov 09, 2011	935	1,085	7.1	7	J	27
SBU-037	Nov 09, 2011	940	1,075	7.1	7	J	2
SBU-038	Nov 09, 2011	997	1,145	9.9	8	J	9
SBU-039	Nov 09, 2011	1,024	1,188	9.2	8	SA	1
SBU-040	Nov 09, 2011	1,046	1,195	10.3	8	SA	11
SBU-041	Nov 09, 2011	1,225	1,370	15.8	10	SA	52
SBU-042	Nov 09, 2011	1,343	1,580	26.3	12	A	8
SBU-043	Nov 09, 2011	1,640	1,780	32.8	17	A	19
SBU-044	Nov 10, 2011	773	918	3.7	5	J	22
SBU-045	Nov 10, 2011	796	931	3.9	5	J	11
SBU-046	Nov 10, 2011	839	945	4.8	6	J	19
SBU-047	Nov 10, 2011	864	985	4.9	6	J	29
SBU-048	Nov 10, 2011	904	1,050	6.8	6	J	19
SBU-049	Nov 10, 2011	914	1,047	6.2	7	J	23
SBU-050	Nov 10, 2011	943	1,093	6.8	7	J	87
SBU-051	Nov 10, 2011	1,124	1,287	12.7	9	SA	2
SBU-052	Nov 10, 2011	1,213	1,363	14.6	10	SA	7

SBU-053	Nov 10, 2011	1,264	1,444	18.1	11	SA	22
SBU-054	May 02, 2012	769	885	4.3	5	J	14
SBU-055	May 02, 2012	778	884	3.4	5	J	18
SBU-056	May 02, 2012	781	891	4.6	5	J	15
SBU-057	May 02, 2012	795	926	1.9	5	J	4
SBU-058	May 02, 2012	815	956	5.3	6	J	10
SBU-059	May 02, 2012	815	940	5.3	6	J	2
SBU-060	May 02, 2012	823	928	5.4	6	J	3
SBU-061	May 02, 2012	860	995	5.9	6	J	109
SBU-062	May 02, 2012	860	998	6.0	6	J	72
SBU-063	May 02, 2012	887	1,022	6.2	6	J	28
SBU-064	May 02, 2012	896	1,029	6.2	6	J	5
SBU-065	May 02, 2012	906	1,060	6.3	6	J	18
SBU-066	May 02, 2012	907	1,053	7.0	6	J	32
SBU-067	May 02, 2012	951	1,093	7.8	7	J	38
SBU-068	May 02, 2012	961	1,098	7.5	7	J	1
SBU-069	May 02, 2012	972	1,110	9.4	7	J	69
SBU-070	May 02, 2012	1,030	1,178	10.4	8	SA	31
SBU-071	May 02, 2012	1,034	1,195	10.2	8	SA	1
SBU-072	May 02, 2012	1,140	1,280	14.9	9	SA	27
SBU-073	May 03, 2012	760	836	3.7	5	J	31
SBU-074	May 03, 2012	769	886	3.7	5	J	11
SBU-075	May 03, 2012	792	928	4.3	5	J	5
SBU-076	May 03, 2012	814	944	5.2	5	J	18
SBU-077	May 03, 2012	819	948	5.3	6	J	60
SBU-078	May 03, 2012	823	945	5.5	6	J	4
SBU-079	May 03, 2012	828	959	5.7	6	J	43
SBU-080	May 03, 2012	860	1,000	6.7	6	J	14
SBU-081	May 03, 2012	861	1,014	5.8	6	J	60

SBU-082	May 03, 2012	861	968	5.7	6	J	1
SBU-083	May 03, 2012	864	993	6.0	6	J	1
SBU-084	May 03, 2012	900	1,066	7.2	6	J	36
SBU-085	May 03, 2012	909	1,052	6.6	7	J	21
SBU-086	May 03, 2012	926	1,062	7.7	7	J	73
SBU-087	May 03, 2012	936	1,076	7.4	7	J	25
SBU-088	May 03, 2012	947	1,089	6.9	7	J	16
SBU-089	May 03, 2012	967	1,132	8.0	7	J	22
SBU-090	May 03, 2012	975	1,136	9.8	7	J	29
SBU-091	May 03, 2012	1,021	1,166	9.4	8	SA	64
SBU-092	May 03, 2012	1,022	1,198	11.3	8	SA	26
SBU-093	May 03, 2012	1,069	1,234	11.6	8	SA	14
SBU-094	May 03, 2012	1,152	1,328	15.1	9	SA	40
SBU-095	May 03, 2012	1,161	1,299	16.4	10	SA	4
SBU-096	May 03, 2012	1,450	1,705	27.0	14	A	31
SBU-097	May 04, 2012	889	1,017	6.6	6	J	35
SBU-098	May 04, 2012	1,022	1,165	10.6	8	SA	5
SBU-099	May 04, 2012	1,316	1,419	28.5	12	A	90
SBU-100	May 28, 2014	718	815	4.0	5	J	8
SBU-101	May 28, 2014	815	915	4.7	6	J	24
SBU-102	May 28, 2014	815	922	4.6	6	J	10
SBU-103	May 28, 2014	825	905	5.0	6	J	1
SBU-104	May 28, 2014	868	998	6.3	6	J	5
SBU-105	May 28, 2014	890	985	6.6	6	J	33
SBU-106	May 28, 2014	918	1,037	6.6	7	J	22
SBU-107	May 28, 2014	982	1,077	9.4	7	J	17
SBU-108	May 28, 2014	1,024	1,165	9.9	8	SA	13
SBU-109	May 28, 2014	1,054	1,201	10.3	8	SA	11
SBU-110	May 28, 2014	1,063	1,232	10.9	8	SA	12

SBU-111	May 28, 2014	1,078	1,225	11.7	9	SA	4
SBU-112	May 28, 2014	1,144	1,305	15.6	9	SA	56
SBU-113	May 29, 2014	827	943	5.2	6	J	5
SBU-114	May 29, 2014	948	1,103	8.0	7	J	16
SBU-115	May 29, 2014	954	1,088	7.6	7	J	10
SBU-116	May 19, 2015	724	849	3.2	5	J	9
SBU-117	May 19, 2015	738	856	3.0	5	J	33
SBU-118	May 19, 2015	770	915	4.0	5	J	310
SBU-119	May 19, 2015	792	906	3.9	5	J	58
SBU-120	May 19, 2015	819	941	4.4	6	J	271
SBU-121	May 19, 2015	820	957	4.5	6	J	7
SBU-122	May 19, 2015	827	969	4.7	6	J	20
SBU-123	May 19, 2015	829	956	4.8	6	J	24
SBU-124	May 19, 2015	838	996	5.0	6	J	52
SBU-125	May 19, 2015	842	986	4.7	6	J	90
SBU-126	May 19, 2015	860	991	5.6	6	J	167
SBU-127	May 19, 2015	863	993	5.3	6	J	42
SBU-128	May 19, 2015	868	1,003	6.3	6	J	61
SBU-129	May 19, 2015	881	1,008	6.1	6	J	95
SBU-130	May 19, 2015	894	1,051	6.7	6	J	9
SBU-131	May 19, 2015	901	1,050	6.4	6	J	16
SBU-132	May 19, 2015	906	1,051	6.8	6	J	22
SBU-133	May 19, 2015	918	1,069	7.2	7	J	42
SBU-134	May 19, 2015	924	1,080	6.6	7	J	19
SBU-135	May 19, 2015	929	1,070	7.0	7	J	4
SBU-136	May 19, 2015	934	1,094	7.4	7	J	47
SBU-137	May 19, 2015	944	1,001	7.3	7	J	16
SBU-138	May 19, 2015	950	1,119	7.4	7	J	63
SBU-139	May 19, 2015	953	1,115	7.2	7	J	7

SBU-140	May 19, 2015	1,004	1,192	9.9	8	SA	63
SBU-141	May 19, 2015	1,080	1,255	11.4	9	SA	73
SBU-142	May 19, 2015	1,153	1,280	12.7	9	SA	1

Note: J = juvenile (500–1,000 mm FL); SA = sub-adult (1,000–1,300 mm FL); A = adult (> 1,300 mm FL); based on National Marine Fisheries Service permitting definitions.

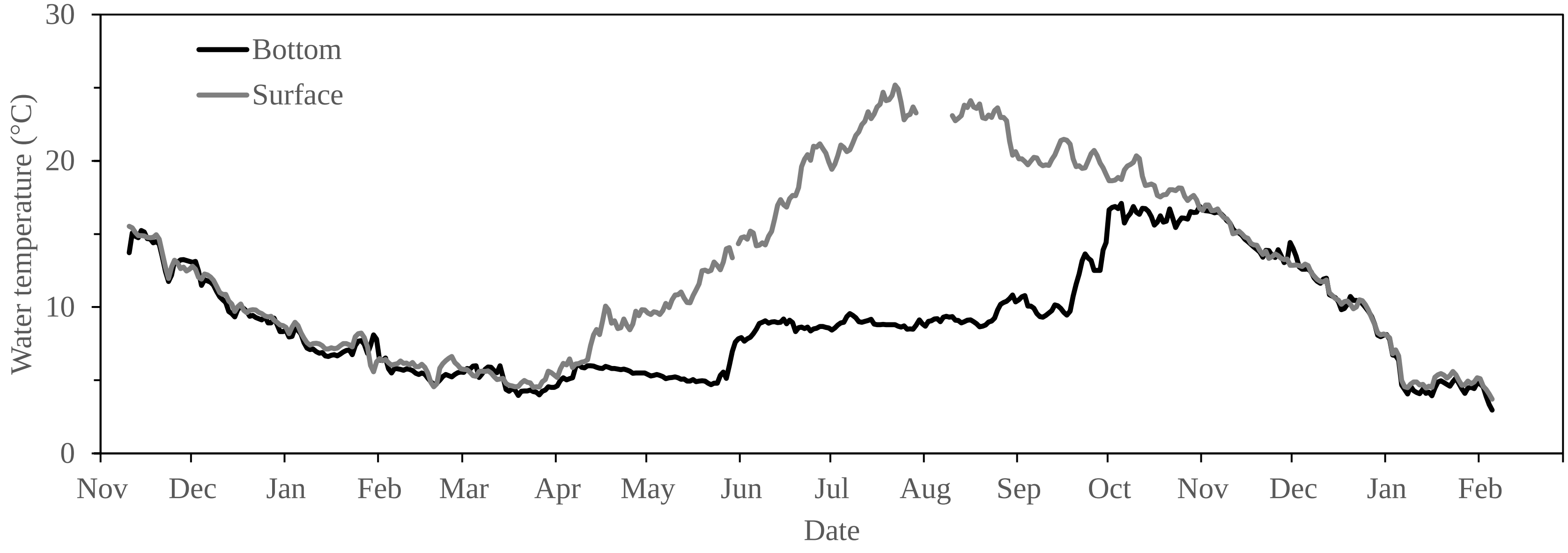

Supplementary Figure S2.—Water temperature in surface and bottom waters of the New York Wind Energy Area study site (Equinor, Lease OCS-A 0512) from November 10, 2016 to February 5, 2018. Bottom temperature data are from transceiver metadata; surface temperature data are from NOAA's NDBC Station 44025.

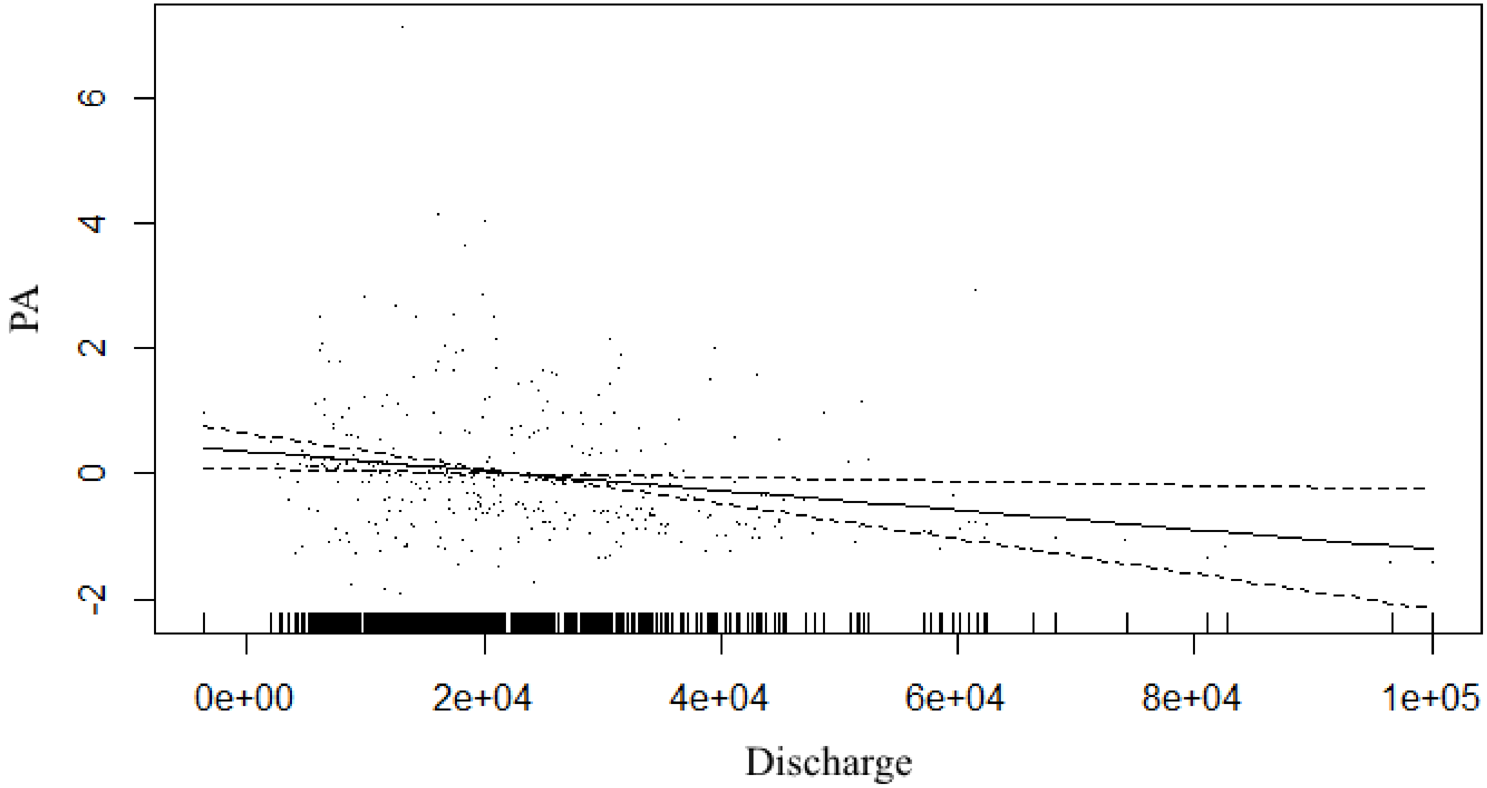

Supplementary Figure S3.—Partial generalized additive model plot identifying the additive effect of Hudson River discharge (ft^3/s) on the probability of occurrence of Atlantic Sturgeon in the New York Wind Energy Area study (PA; NY WEA; Equinor, Lease OCS-A 0512). The range of the *y* axis is indicative of the relative importance of the covariate. The *x* axis reflects the relative density of data points as shown by the "rug." Dashed lines reflect the 95% confidence intervals around response curves. Discharge is from the USGS Gauging Station 01372058 at Hudson River below Poughkeepsie, New York.

References

1. von Bertalanffy, L. A quantitative theory of organic (inquiries on growth laws, II). *Hum. Biol.* **10,** 181-213 (1938).

2. Dunton, K. J. *et al.* Age and growth of Atlantic Sturgeon in the New York Bight. *N. Am. J. Fish. Manag.* **36,** 62–73 (2016).
